# 87. The safety and efficacy advantage of blocking C5a *vs* C5 in critically ill, COVID-19 patients: Results from PANAMO, a Phase 3 randomized controlled trial

**DOI:** 10.1093/ofid/ofad500.003

**Published:** 2023-11-27

**Authors:** Bruce P Burnett, Endry H T Lim, Alexander Vlaar, Sanne De Bruin, Matthijs Brouwer, Maria Habel, Claus Thielert, James Dickinson, simon Rückinger, Robert Zerbib, Dorothee Neukirchen, Renfeng Guo, Diederik van de Beek, Niels Riedemann

**Affiliations:** InflaRx GmbH, InflaRx Pharmaceuticals, Inc., Fuquay Varina, NC; Amsterdam UMC, Amsterdam, Noord-Holland, Netherlands; Amsterdam UMC, Amsterdam, Noord-Holland, Netherlands; Amsterdam UMC, Amsterdam, Noord-Holland, Netherlands; Amsterdam UMC, Amsterdam, Noord-Holland, Netherlands; InflaRx GmbH, Jena, Thuringen, Germany; InflaRx GmbH, Jena, Thuringen, Germany; InflaRx GmbH, Jena, Thuringen, Germany; Metranomia Clinical Research GmbH, Munich, Bayern, Germany; InflaRx GmbH, Jena, Thuringen, Germany; InflaRx GmbH, Jena, Thuringen, Germany; InflaRx GmbH, Jena, Thuringen, Germany; Amsterdam UMC, Amsterdam, Noord-Holland, Netherlands; InflaRx GmbH, Jena, Thuringen, Germany

## Abstract

**Background:**

C5 convertase cleaves C5 in common complement pathways yielding the anaphylatoxin C5a and C5b, part of the membrane attack complex (MAC) that mitigates bacterial infections (Fig 1). C5 can also be cleaved “extrinsically” by other enzymes. C5a is elevated in severe COVID-19 patients (Pt). Yet, inhibiting C5 cleavage increases infections without improved survival in critically ill COVID-19 Pt (Annane et al. 2023). In addition, eculizumab, a C5 blocker does not prevent C5a from being generated in COVID-19 Pt (Raghunandan et al. 2020). Vilobelimab (Vilo), an anti-C5a monoclonal antibody that preserves MAC, was tested in a Phase 3 multicenter, double-blind, randomized, placebo (Plc)-controlled study for its effect on survival in critically ill COVID-19 Pt (Vlaar et al. 2023).

**Methods:**

COVID-19 Pt recruited worldwide (N=368; Vilo n=177, Plc n=191) intubated within 48 hrs before treatment, received up to 6, 800mg infusions of Vilo or Plc on top of standard-of-care. The primary endpoint was 28-Day all-cause mortality with key secondary endpoints of 60-Day mortality and safety. Blood samples were assessed by ELISA at screening, Day 8 and at hospital discharge for Vilo and C5a levels. No meningococcal vaccination or prophylactic antibiotics were used in the study.

**Figure:**
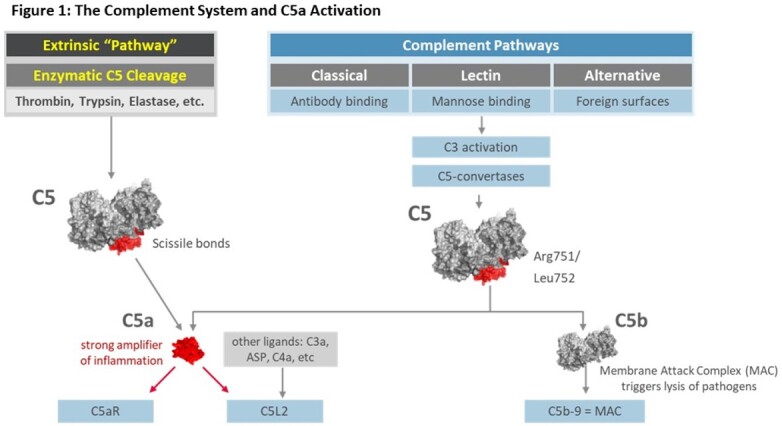

**Results:**

Kaplan-Meier estimates showed a 28-Day mortality rate of 31.7% for Vilo *vs* 41.6% for Plc [HR 0.67 (95% CI: 0.48, 0.96), p< 0.05] with similar 60-Day results. On Day 8 after 3 infusions, mean Vilo trough levels were 21,799.3 to 302,972.1 ng/mL (geometric mean 137,881.3 ng/mL). C5a was highly elevated and comparable between groups at screening: Vilo mean 130.3 ng/mL, Plc mean 123.2 ng/mL. By Day 8, C5a levels were reduced 87% for Vilo (mean 16.8 ng/mL, p< 0.001) *vs* an increase for Plc (mean 129.8 ng/mL). Though post Day 8 sampling was sparse, C5a levels remained elevated for Plc and low for Vilo throughout the study. Serious AEs were 58.9% for Vilo and 63.5% for Plc. Treatment-emergent AEs were 90.9% for Vilo *vs* 91.0% for Plc. Infection incidence per 100 Pt days was comparable in both groups.

**Conclusion:**

Results from this Phase 3 study show that direct C5a inhibition by vilobelimab while presumably preserving MAC, as opposed to upstream C5 blockade, results in a survival benefit for critically ill COVID-19 Pt without increasing infections.

**Disclosures:**

**Bruce P. Burnett, PhD**, InflaRx GmbH, InflaRx Pharmaceuticals, Inc.: Employee **Alexander Vlaar, MD, PhD**, InflaRx: Advisor/Consultant **Maria Habel, PhD**, InflaRx GmbH: Employee **Claus Thielert, PhD**, InflaRx GmbH: Employee **James Dickinson, MSc**, InflaRx GmbH: Employee **simon Rückinger, PhD**, Metronomia Clinical Research GmbH: Advisor/Consultant **Robert Zerbib, MSc**, InflaRx GmbH: Advisor/Consultant **Dorothee Neukirchen, PhD**, InflaRx GmbH: Employee **Renfeng Guo, MD**, inflarx: Board Member|inflarx: I am an Inventor for Vilobelimab|inflarx: Ownership Interest|inflarx: Stocks/Bonds|InflaRx GmbH, InflaRx Pharmaceuticals, Inc.: Board Member|InflaRx GmbH, InflaRx Pharmaceuticals, Inc.: Founder|InflaRx GmbH, InflaRx Pharmaceuticals, Inc.: Employee|InflaRx GmbH, InflaRx Pharmaceuticals, Inc.: Ownership Interest **Niels Riedemann, MD, PhD**, InflaRx GmbH, InflaRx Pharmaceuticals, Inc.: Board Member|InflaRx GmbH, InflaRx Pharmaceuticals, Inc.: Founder|InflaRx GmbH, InflaRx Pharmaceuticals, Inc.: Employee|InflaRx GmbH, InflaRx Pharmaceuticals, Inc.: Ownership Interest

